# The Short Treatment Allocation Tool for Eating Disorders: current practices in assigning patients to level of care

**DOI:** 10.1186/s40337-018-0230-2

**Published:** 2018-12-19

**Authors:** Josie Geller, Leanna Isserlin, Emily Seale, Megumi M. Iyar, Jennifer S. Coelho, Suja Srikameswaran, Mark Norris

**Affiliations:** 1 Eating Disorders Program, St. Paul’s Hospital, 1081 Burrard Street, Vancouver, British Columbia V6Z 1Y6 Canada; 2 Department of Psychiatry, University of British Columbia, Vancouver, British Columbia Canada; 3 Department of Psychiatry, Children’s Hospital of Eastern Ontario, Ottawa, Ontario Canada; 4 Department of Psychology, University of British Columbia, Kelowna, British Columbia Canada; 5 B.C. Children’s Hospital Provincial Specialized Eating Disorders Program for Children & Adolescents, Vancouver, British Columbia Canada; 6 Department of Pediatrics, University of Ottawa, Children’s Hospital of Eastern Ontario, Ottawa, Canada

**Keywords:** Eating disorders, Treatment allocation, Decision-making, Readiness, Assessment, Evidence-based practice, Guidelines

## Abstract

**Objective:**

The Short Treatment Allocation Tool for Eating Disorders (*STATED)* is a new evidence-based algorithm developed to match patients to the most clinically appropriate and cost-effective level of care (Geller et al., 2016). The objective of this research was to examine the extent to which current practices are in alignment with *STATED* recommendations.

**Method:**

Participants were 179 healthcare professionals providing care for youth and/or adults with eating disorders. They completed an online survey and rated the extent to which three patient dimensions (*medical stability*, *symptom severity*, and *readiness*) were used in assigning patients to each of five levels of care*.*

**Results:**

The majority of analyses testing a priori hypotheses based on the *STATED* were statistically significant (all *p*’s < .001), in the direction of *STATED* recommendations. However, a strict coding scheme evaluating the extent to which ratings were fully consistent with the *STATED* showed inconsistency rates ranging from 17 to 55% across the five levels of care, with the greatest inconsistencies involving the use of *readiness* information, and the lowest involving the use of *medical stability* information.

**Discussion:**

Although practices were generally aligned with the *STATED* recommendations*, readiness* information was used least consistently in assigning patients to level of care.

## Plain English summary

The Short Treatment Allocation Tool for Eating Disorders (*STATED)* is a new tool developed to help match patients to the most appropriate and cost-effective care (Geller et al., 2016). The objective of this research was to determine if current practices are in alignment with *STATED* recommendations. Healthcare professionals providing care for youth and/or adults with eating disorders completed an online survey and rated the extent to which they used the *STATED* guidelines to assign patients to the appropriate level of care. It was determined that although practices were generally aligned with the *STATED* recommendations*,* there were some inconsistencies, with information about readiness for change being used least reliably in assigning patients to level of care.

## Background

Eating disorders (EDs) are often chronic conditions characterized by treatment refusal, premature termination, and relapse across all levels of care resulting in significant health care costs [1]. There is little consensus regarding which patient characteristics are most helpful in assigning patients to the most appropriate level of care. For instance, although there is general agreement that hospitalization is indicated for a medically unstable patient, there is little consensus about how other patient factors should inform assignment to treatment, such as outpatient, day, or residential care. Despite a robust literature showing that readiness and motivation to recover is one of the strongest predictors of clinical outcome [2], this evidence is not systematically included in current decision-making models [3].

The Short Treatment Allocation Tool for Eating Disorders (*STATED)* is a simple innovative evidence-based algorithm that uses three patient dimensions; *medical stability*, *symptom severity/life interference*, and *readiness/engagement* in assigning level of care for individuals with EDs [3]. The *STATED* is unique in its inclusion of readiness information and in its allowance of independent variations along the three continua. Readiness refers to an individual’s internal motivation to engage in symptom reduction goals of action-oriented treatment. The *STATED* was developed in the context of the Canadian health care system where there are five levels of publically funded resources: two lesser resource outpatient options, two higher resource options involving a combination of outpatient residential and inpatient settings and finally, inpatient hospital admission (see Table 1). These levels of care are similar to those outlined in the American Psychiatric Association [4] guidelines, with the exception that only the *STATED* includes a treatment option that focuses on quality of life, recommended and shown to be effective for individuals whose readiness is low and whose symptom severity is high [5, 6].

**Table 1:**
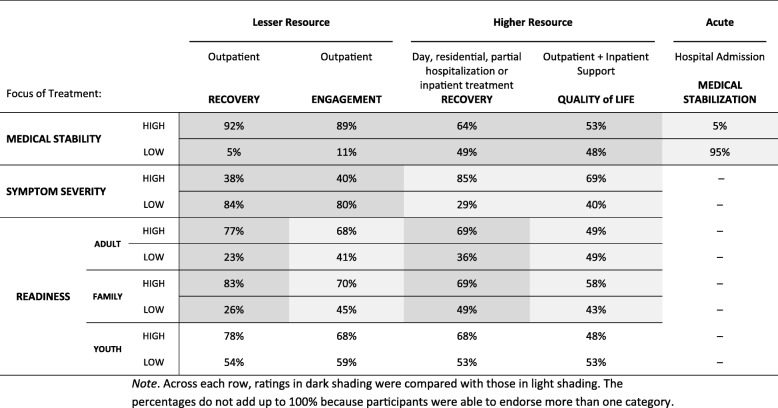
Allocation of Patients According to the STATED Dimensions (%)

The *STATED* was developed with the intent to promote best resource utilization in its use of empirical evidence in matching patients to treatment [3]. In its inclusion of readiness as a central component, the *STATED* is supported by two decades of research showing the key role that readiness plays in predicting symptom improvement, dropout and relapse in this population [7–10]. The development of the *STATED* came about in response to previous protocols either failing to take into account patient readiness for treatment and/or assumptions about patients (i.e., high readiness co-occurs with low symptom severity) that don’t reflect real-world, clinical experience.

**Fig. 1:**
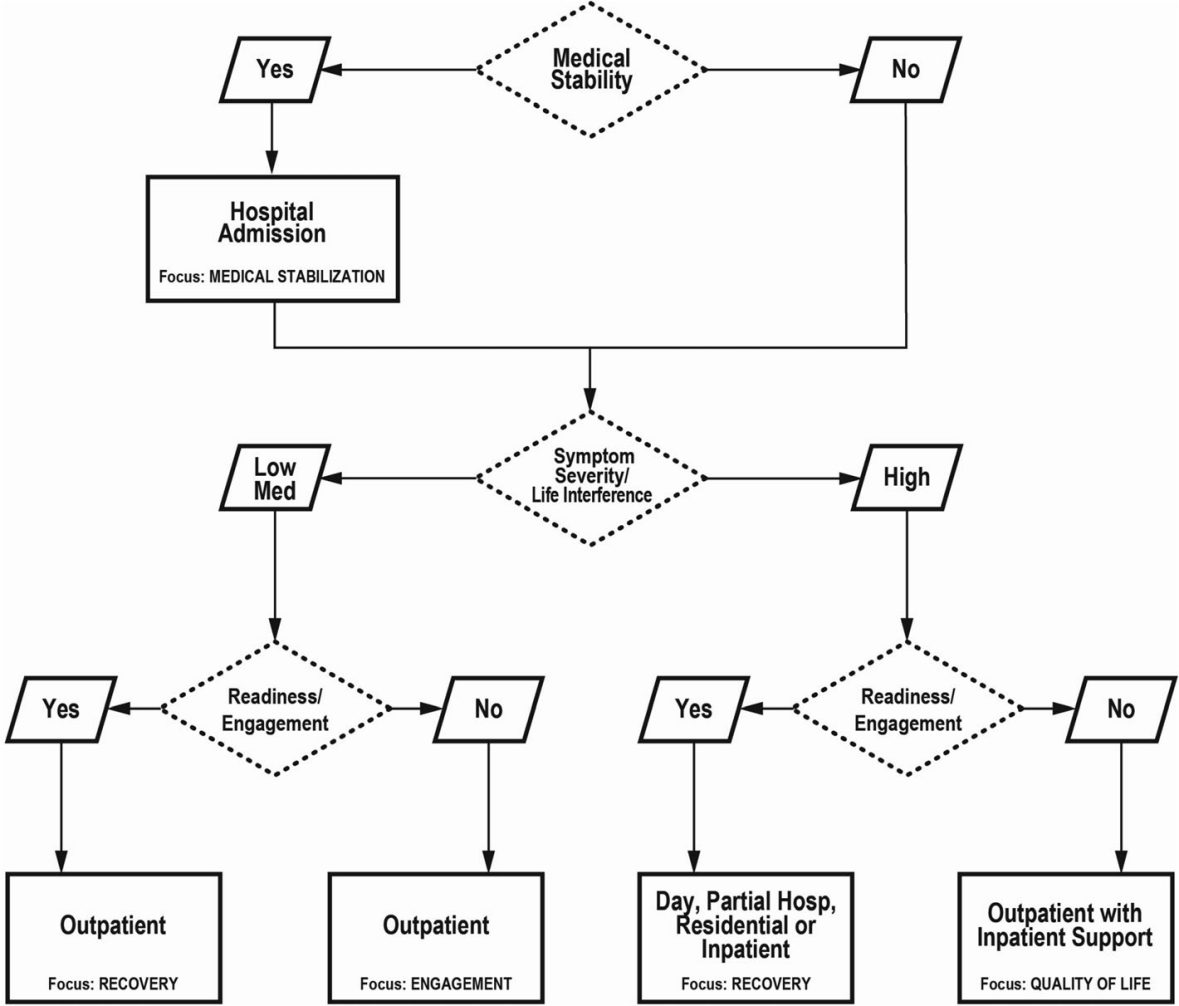
Application of the STATED. Reproduced from Geller et al., 2016

The *STATED* is trans-diagnostic and represents all patient presentations across the developmental spectrum. Figure 1 depicts how patient information is used to assign patients to level of care. As shown, *medical stability*, defined as a patient’s immediate medical risk (“yes” indicates low risk, “no” indicates risk, or medical instability), is the only information needed to determine whether a patient requires a hospital admission. *Symptom severity* is used to determine whether a patient requires higher resource (i.e., day, residential or inpatient treatment or outpatient with inpatient support) vs. lesser resource (i.e., outpatient) care. Finally, *readiness* is used within higher and lesser resource treatment options, to determine the focus of treatment. That is, within lesser resource outpatient treatment, the focus for patients with high readiness is on recovery (e.g., Cognitive Behaviour Therapy-Enhanced; CBT-E or Family Based Therapy; FBT), vs. engagement for low readiness patients. Conversely, for higher resource options, the focus for patients with high readiness is on recovery (day, residential or inpatient treatment) vs. on quality of life (outpatient with inpatient support) for patients with low readiness. For children and youth, the use of readiness information depends upon both therapeutic modality and characteristics of the youth/family. For example, for children and young adolescents, parents’ readiness may be most central to determining the potential appropriateness of recovery-based outpatient treatments (such as FBT). For older adolescents, a mix of assessment of readiness of youth and family may be considered. Given the potential for irreversible medical complications, the level of care focusing on quality of life is less likely to be recommended for pediatric populations. The *STATED* assesses similar characteristics to other classification systems (e.g., APA guidelines), but allows for flexibility in how the dimensions co-occur. For instance, two dimensions identified in the APA guidelines, psychiatric comorbidities and suicidality, would contribute to the symptom severity or medical acuity dimensions of the *STATED*, depending upon their severity.

Little is known about the use of empirical evidence in assigning patients with EDs to treatment. The purpose of this research was to determine the extent to which current allocation of patients to level of care is in alignment with *STATED* recommendations.

## Methods

Letters describing the study were sent via email to ED organization listservs (e.g., Academy for Eating Disorders, Eating Disorders Research Society, Eating Disorders Association of Canada) for distribution. The survey remained open for three months and potential participants were encouraged to pass on study information to others to increase the dissemination of materials. The secure online Research Electronic Data Capture (REDCap) web-based platform was used for development and administration [11]. The study received Research Ethics Board approval and all participants provided informed consent.

### Participants

Healthcare professionals who self-identified as providing care for youth and/or adults with EDs were eligible to participate. Three hundred and sixteen initiated the online survey, of which 179 (56.6%) completed all questions and were retained for analysis. The majority of participants were female (*n* = 153, 86%). Almost 95% of respondents resided either in Canada (*n* = 116, 65%) or the United States (*n* = 51, 29%), with the remaining participants practicing in ten other countries. The majority of participants were psychologists (*n* = 47) followed by physicians (*n* = 40), nutritionists and registered dieticians (*n* = 27), therapists (*n* = 23), social workers (*n* = 20), nurses (*n* = 12), occupational therapists (*n* = 5) and other (*n* = 5). Thirty-seven percent of participants worked in centers with intensive treatment programs including inpatient or residential facilities (*n* = 67), 31% in outpatient treatment settings that were affiliated with a larger ED team or network (*n* = 55) and 31% worked in private practice (*n* = 55). Forty-six percent of participants (*n* = 83) worked in a practice that was affiliated with an academic institution. The length of time that participants had been working in eating disorders field was: less than 5 years (*n* = 53, 30%), 6–15 years (*n* = 71, 40%), and 16 years or more (*n* = 55, 31%).

### *STATED* survey

The *STATED* survey was developed as part of a larger research study by six senior Canadian clinicians working in the ED field. It was piloted within two tertiary level ED-programs (one adult and one youth), and modifications were made to improve readability and clarity.

The survey consisted of two sections: Part 1 addressed demographic and background variables, including country of origin, treatment setting, and characteristics of patients treated. In Part 2, participants rated how patient characteristics inform current practices in assigning patients to each of the five levels of care. That is, for each treatment level, participants provided ratings according to three patient dimensions: *medical stability*, *symptom severity*, and *readiness.* The readiness category was further broken down into adult patients, youth patients, and family. Participants were asked to draw upon their experiences and clinical judgment in indicating the extent to which each patient characteristic (e.g., medical acuity), was currently perceived to be appropriate for each level of care using four-point Likert scales [e.g., not applicable (N/A), low, moderate or high]. It was possible to select one or multiple ratings.

### Planned analyses

Two sets of analyses were performed using SPSS version 24.0. First, the pattern of “high” and “low” responses across the five levels of care was examined to determine the extent to which responses were consistent with *STATED* recommendations. We focused our analysis on the end points of each continuum given a) our sample size, b) the majority of *STATED* recommendations being based upon the end points of each continuum and c) the challenge in interpreting “moderate” ratings given the tendency for these responses to be a “dumping ground” for unsure participants [12]. For each dimension (e.g., *medical stability*), Cochran’s *Q* tests were conducted comparing ratings across the levels of care, and when significant, were followed up with alpha-corrected McNemar tests (see Table 1). Thus, eight Cochran’s *Q* tests were performed (*medical stability* High, *medical stability* Low, *symptom severity* High, *symptom severity* Low, *adult readiness* High, *adult readiness* Low, *family readiness* High, and *family readiness* Low).

Alpha corrected McNemar comparisons were conducted to follow up each significant omnibus Cochran’s *Q*. That is, for *medical stability,* four comparisons determined if the “hospital admission” level of care had more “high” than the other four levels of care, with alpha set at .05/4 = .0125. Similarly, four comparisons determined if the “hospital admission” level of care had fewer “low” than the other four levels of care, also with alpha set at .0125. For *symptom severity*, eight McNemar tests were conducted to determine if the two lesser resource outpatient options had less “high” and more “low” severity ratings than the two higher resource options. For *adult and family readiness*, within the lesser and higher resource options, McNemar tests were conducted to determine if the recovery focused levels of care had higher readiness ratings (i.e., more “high” and less “low”) than the engagement and quality of life focused options. Thus, four McNemar tests were conducted for adults, and four for families. Given the dearth of empirical literature on *youth readiness* and the different level of importance assigned to this variable according to patient/family characteristics (including age of the youth), no a priori hypotheses were made based upon *youth readiness*.

The second set of analyses examined the extent to which current practices were consistent with *STATED* recommendations using an a priori binary coding system for each of the 25 *STATED* items, coded as either 1 = “consistent” or 0 = “inconsistent”. *STATED* “inconsistent” responses were coded as follows:

(i) *Medical stability* was “high” or “N/A” for lesser resource options (i.e., recovery or engagement-focused outpatient), “N/A” for higher resource options (i.e., inpatient/day/residential or quality of life focus), and “low” or “N/A” for hospital admission,
(ii) *Symptom severity* was “high” or “N/A” for lesser resource options, and “low” or “N/A” for higher resource options
(iii) *Adult and family readiness* was “low” or “N/A” for the recovery-focused lesser resource options, “low” or “N/A” for the recovery-focused higher resource options, “high” or “N/A” for the engagement-focused lesser resource options and the quality of life higher resource options.

## Results

### Allocation of patients according to *STATED* dimensions

Table 1 describes the proportion of participants who provided “high” and “low” ratings for *medical stability*, *symptom severity* and *readiness* across the levels of care. As shown in the table, across each row, dark shaded ratings were compared with those with light shading. A Cochran’s *Q* test was conducted for each of the eight shaded rows of the table. All were significant (*medical stability* high, *Q* [4] = 379.06, *p* < .001; *medical stability* low, *Q* [4] = 370.83, *p* < .001; *symptom severity* high, *Q* [3] = 144.95, *p* < .001; *symptom severity* low, *Q* [3] = 202.69, *p* < .001; *adult readiness* high, *Q* [3] = 60.09, *p* < .001; *adult readiness* low, *Q* [3] = 33.79, *p* < .001; *family readiness* high, *Q* [3] = 52.18, *p* < .001; *family readiness* low, *Q* [3] = 32.56, *p* < .001).

McNemar follow-up tests were conducted to determine whether the significant differences detected between levels of care matched recommendations made by the *STATED*. Each set of McNemar analyses used an alpha-corrected significance level to control for the number of comparisons (see Analysis Plan). Of the 24 tests conducted, 22 were statistically significant (all *p*’s < .001) and consistent with *STATED* recommendations. The two non-significant comparisons were for *family readiness*, with “high” ratings for outpatient recovery vs. outpatient engagement, and *family readiness*, “low” ratings for intensive recovery focused treatment vs. quality of life focus outpatient with inpatient support options.

### *STATED* inconsistent responses

Using the binary coding system, the proportion of *STATED* inconsistent responses was calculated for each of the three *STATED* dimensions and across the five levels of care. The proportion of “inconsistent” responses across the three *STATED* dimensions was: *medical stability* (9%), *symptom severity* (40%), *adult readiness* (58%), and *family readiness* (66%). The proportion of *STATED* inconsistent responses across the five levels of care was: outpatient recovery-focused (30%), outpatient engagement-focused (55%), recovery-focused intensive day, residential or inpatient care (38%), quality of life-focused outpatient care with inpatient support (48%), and hospital admission for medical stabilization (17%).

## Discussion

The objective of this research was to examine the extent to which current practices are in alignment with the *STATED*. Overall, results supported a trend for practices to be in agreement with *STATED* recommendations*,* with 22 out of 24 comparisons reaching statistical significance in the direction of a priori hypotheses*.* Patients who had low *medical stability* were seen as more suited for hospital medical stabilization treatment than for other less intensive forms of care. Patients with higher *symptom severity* were seen as more suited for higher resource day, inpatient, or residential treatment or quality of life focused treatment as opposed to other outpatient treatment options. Finally, across lesser and higher resource options, patients with higher *readiness* were seen as more suited for recovery-focused treatment. These findings suggest that the practices in this sample of clinicians were overall in alignment with *STATED* recommendations.

Using a more stringent coding system, however, despite an overall trend for agreement, high levels of inconsistency were nevertheless detected, particularly in the *readiness* dimension (58% for adults and 66% for families). Possible explanations for the inconsistency for families include a lack of understanding of the implications of low readiness, the absence of validated measures, and the paucity of research on family readiness. It is also possible that lack of availability of alternatives to action-oriented treatment, such as care focusing on quality of life for individuals who are very ill and whose readiness is low was also a factor. Finally, *STATED* inconsistent responses may occur in an effort to relieve distress by offering care for patients low in readiness. For instance, patients with low readiness were frequently assigned to recovery-focused intensive day, residential or inpatient care. The lowest levels of “inconsistent” responses were detected for the *medical stability* dimension, likely due to the universal recognition of the critical need to assess medical stability and to prevent de-compensation and death in critically ill patients.

There are several limitations to this research. First, although this study identified the extent to which current practices are in alignment with *STATED* recommendations, factors contributing to alignment were not explored. For instance, it is not known whether participants’ backgrounds, prior knowledge of the *STATED,* or geographical location (and corresponding access to, or familiarity with different levels of care), contributed to *STATED* consistent responses. Another limitation of the research is the relatively low completion rate of individuals who activated the survey (57%). Drop-out rates prior to study completion of web-based surveys have been shown to vary widely, ranging from 0 to 73% [13]. Factors shown to be associated with lower completion rates in web-based surveys that may be relevant to this research include one-time respondents vs. specific targeting of individuals and a larger, vs. smaller number of contacts [14]. Another possible concern is that our use of categorical coding schemes decreased sensitivity. The large number of significant findings (22/24) however, suggests that lack of sensitivity was not an issue. Finally, our sample was comprised primarily of clinicians from North America and generalizability to other countries is not known.

## Conclusion

The *STATED* uses three evidence-based dimensions for decision-making regarding treatment allocation to maximize benefits to patients and families and make the most efficient use of health care resources. This research suggests that while clinicians are generally consistent in their use of the *STATED* framework, future research is needed to understand factors contributing to challenges in using readiness information in treatment allocation. For example, we need to help patients and families understand that action-oriented treatment is unhelpful when patients/families do not see themselves as having a problem, or in other words, have low readiness. Building awareness amongst patients and families about the importance of readiness is a first step. Amongst clinicians, one possible means to improve the assessment of readiness is providing training to assessors in the use of a collaborative/ motivational interviewing style to ensure accurate assessment of patient and family readiness. Additionally, it would be helpful to ensure that a menu of treatment options is available with clear program guidelines outlining rationale and expectations for each level of care. It is thus hoped that the *STATED* framework supports collaborative discussions between patients and care providers in which the rationale for matching patient characteristics to levels of care is transparent, and that allows decisions to be made that are most suited to patient’s wishes and health care needs.

## Abbreviations

APA: American Psychiatric Association
CBT-E: Cognitive Behaviour Therapy-Enhanced
ED: Eating disorders
FBT: Family Based Therapy
N/A: Not applicable
REDCap: Research Electronic Data Capture
STATED: The Short Treatment Allocation Tool for Eating Disorders
